# Objectively measured physical activity was not associated with neighborhood walkability attributes in community-dwelling patients with stroke

**DOI:** 10.1038/s41598-022-07467-y

**Published:** 2022-03-03

**Authors:** Masashi Kanai, Kazuhiro P. Izawa, Hiroki Kubo, Masafumi Nozoe, Shinichi Shimada

**Affiliations:** 1 Department of Physical Therapy, Faculty of Nursing and Rehabilitation, Konan Women’s University, 6-2-23 Morikitamachi, Higashinada-ku, Kobe, 658-0001 Japan; 2 Department of Public Health, Graduate School of Health Sciences, Kobe University, 7-10-2 Tomogaoka, Suma-ku, Kobe, 654-0142 Japan; 3 Cardiovascular Stroke Renal Project (CRP), 7-10-2 Tomogaoka, Suma-ku, Kobe, 654-0142 Japan; 4 Department of Rehabilitation, Itami Kousei Neurosurgical Hospital, 1-300-1 Nishino, Itami, 664-0028 Japan; 5 Department of Neurosurgery, Itami Kousei Neurosurgical Hospital, 1-300-1 Nishino, Itami, 664-0028 Japan

**Keywords:** Stroke, Lifestyle modification, Public health

## Abstract

Although the built environment may affect physical activity, there is little evidence on how neighborhood walkability attributes influence post-stroke physical activity. This study aimed to explore associations between objectively measured physical activity and neighborhood walkability attributes in community-dwelling patients with stroke. This cross-sectional study recruited patients who could ambulate outside free of assistance. We assessed objectively measured physical activity comprising the number of steps taken and time spent in moderate-to-vigorous physical activity (MVPA) with an accelerometer. Neighborhood walkability attributes were evaluated using the Walk Score. Multiple linear regression analyses were used to determine whether the Walk Score was independently associated with the number of steps taken or MVPA. Eighty participants with a mean age of 65.9 ± 11.1 years were included. The participants took an average of 5900.6 ± 2947.3 steps/day and spent an average of 19.7 ± 21.7 min/day in MVPA. The mean Walk Score was 71.4 ± 17.2. Multiple linear regression analyses showed that no significant associations were found between the Walk Score and the number of steps taken or MVPA. No associations were found between objectively measured physical activity and neighborhood walkability attributes in community-dwelling patients with stroke in an Asian area.

## Introduction

It is well known that physical activity after stroke is a major target to prevent not only stroke recurrence^1,2^ but also all-cause mortality^3^. In a randomized controlled trial, Kono et al. reported that lifestyle interventions, including the promotion of physical activity, in patients with mild stroke led to a reduction in new vascular events, including recurrent stroke^1^. They also showed that at least approximately 6000 steps per day were appropriate to prevent the occurrence of a new vascular event after stroke^2^. Loprinzi and Addoh indicated in a national prospective cohort study of adults with post-acute stroke that physical activity among stroke survivors is inversely associated with all-cause mortality^3^. For these reasons, studies focusing on the promotion of physical activity itself and studies of the comprehensive prevention of recurrence that include the promotion of physical activity are being conducted^4,5^.

Exercise and physical activity influence, or have the potential to influence, the process of post-stroke disablement at various points^6^. Once a stroke occurs or is recurrent, the cycle of “detraining post-stroke” begins, which means that impairments to physical fitness occur that lead to activity limitations, physical inactivity, and the occurrence of additional impairments to physical fitness^6^. To break this negative loop, it may be necessary to focus on instruction beyond that related to physical functions. Thilarajah et al. reported that physical function only accounted for half of the variance in post-stroke physical activity levels in their systematic review and that several modifiable and non-modifiable factors are associated with post-stroke physical activity^7^.

Although the determination of physical activity is multifaceted and complex, ecological models emphasize the role played by the built environment in supporting an active lifestyle^8^. We previously reported that the average number of steps was significantly associated with perceived built environment attributes such as the presence of a sidewalk and access to recreational facilities in community-dwelling ambulatory patients with stroke^9^. However, because this study evaluated built environment attributes by questionnaire, each individual patient might have perceived the neighborhood environment differently. It seems that individuals who regularly engage in physical activity in their neighborhood may have more accurate perceptions and be aware of specific characteristics of their neighborhood^10^. Therefore, objective measures such as a geographic information system^11^ and the Walk Score^12–14^ are also often used to assess the built environment in relation to physical activity. Chen et al. reported that high sidewalk availability, which is one of the neighborhood walkability factors, may be supportive for daily steps in older Asian adults^15^. However, there is little evidence regarding associations between objectively measured physical activity and neighborhood walkability attributes in patients with stroke^16^. If these associations are clarified, we may be able to develop appropriate methods to promote physical activity in patients with stroke depending on their neighborhood walkability attributes.

We thus hypothesized that objectively measured physical activity would be associated with neighborhood walkability attributes in patients with stroke. The purpose of the present study was to determine associations between objectively measured physical activity and neighborhood walkability attributes in community-dwelling patients with stroke.

## Results

**Figure 1:**
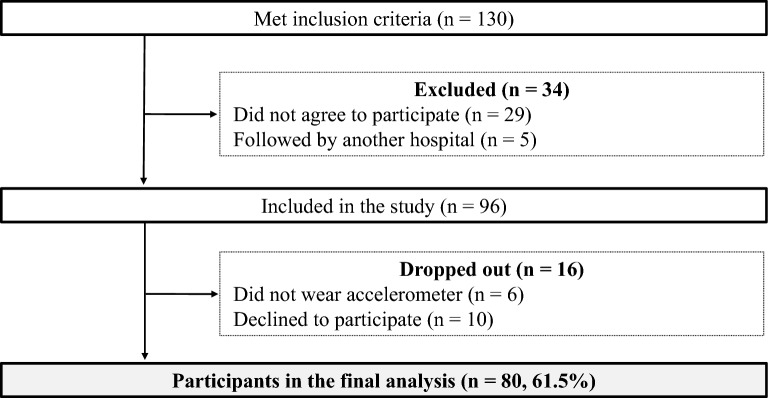
Participant flow in the present study.

Participant flow through the present study is shown in Fig. 1. Of the 130 original patients, 34 patients were excluded because they did not agree to participate (n = 29) or were followed by another hospital (n = 5). Ninety-six patients were included in the study, but 16 patients later dropped out because they did not wear the accelerometer (n = 6) or declined to participate (n = 10). Finally, 80 participants (61.5%) with a mean age of 65.9 ± 11.1 (standard deviation) years were included in the present study. Clinical characteristics of the patients are shown in Table 1.

**Table 1:** Clinical characteristics.

Characteristic	All participants (n = 80)
Age (years)	65.9 ± 11.1
Sex (male), n (%)	58 (72.5)
BMI (kg/m^2^)	23.5 ± 2.8
NIHSS (score)	1.3 ± 0.5
Time since stroke (months)	4.9 ± 4.5
Living situation (not alone), n (%)	68 (85.0)
Working status (employed), n (%)	21 (26.3)
Long-term care insurance, n (%)	25 (31.3)
Walking speed (m/s)	1.3 ± 0.3

Data are expressed as mean ± SD or number (percentage).

*BMI* body mass index, *NIHSS* National Institutes of Health Stroke Scale.

The participants took an average of 5900.6 ± 2947.3 steps/day and spent an average of 19.7 ± 21.7 min/day in moderate-to-vigorous physical activity (MVPA). The mean Walk Score was 71.4 ± 17.2 (range 7 to 97). Based on the Walk Score, five individuals lived in a “car-dependent” area, 29 lived in a “somewhat walkable” area, 40 lived in a “very walkable” area, and six lived in a “walker’s paradise” area.

In the univariate analysis, number of the steps was significantly associated with age, sex, NIHSS, working status, long-term care insurance, and walking speed. MVPA was also significantly associated with these same factors except for age.

Table 2 shows the results of the multiple linear regression analyses. Walk Score was not significantly associated with either the number of steps taken or with MVPA. Only working status was significantly associated with the number of steps taken (standardized β = 0.25, p = 0.046), and only sex was significantly associated with MVPA (standardized β = − 0.24, p = 0.030).

**Table 2:** Associations between objectively measured physical activity and walkability by multiple linear regression analysis.

Variables	Number of steps R^2^ = 0.32 Adjusted R^2^ = 0.23		MVPA R^2^ = 0.25 Adjusted R^2^ = 0.16	
	Standardized β	p value	Standardized β	p value
**Walk Score**				
Q1 least walkable	Reference			
Q2	− 0.12	0.364	− 0.14	0.303
Q3	− 0.04	0.766	− 0.03	0.809
Q4 most walkable	− 0.07	0.604	− 0.04	0.782
Age	− 0.06	0.590		
Sex, female	− 0.18	0.082	− 0.24	0.030
NIHSS	0.01	0.965	0.04	0.787
Working status	0.25	0.046	0.09	0.432
Long-term care insurance	− 0.19	0.132	− 0.20	0.145
Walking speed	0.15	0.247	0.19	0.170

*MVPA* moderate-to-vigorous physical activity, *NIHSS* National Institutes of Health Stroke Scale, *R*^*2*^ coefficient of determination, *β* regression coefficient.

## Discussion

Contrary to our hypothesis, the present study found no association between objectively measured physical activity such as the number of the steps taken or MVPA and neighborhood walkability attributes in community-dwelling patients with stroke. Alternatively, working status was significantly associated with the number of steps, and sex was significantly associated with MVPA in this population.

In the present study, the average number of steps taken by the patients with stroke was 5900.6 ± 2947.3 steps/day. A systematic review reported that subacute stroke patients took an average of 5535 steps/day, and chronic stroke patients took an average of 4078 steps/day^17^. Although post-stroke physical activity depends on the severity of the stroke or time since stroke onset, the number of steps taken in the present study was equivalent to that of the mean value of the studies investigated in the systemic review by Fini et al.^17^, in which a variety of different measurement devices were used. The participants in the present study spent an average of 19.7 ± 21.7 min/day in MVPA. New guidelines for the prevention of stroke in patients with stroke and transient ischemic attack from the American Heart Association/American Stroke Association recommend that patients who are capable of physical activity engage in at least moderate-intensity aerobic activity for a minimum of 10 min 4 times a week or vigorous-intensity aerobic activity for a minimum of 20 min twice a week^18^. Although we could not be certain how often the participants in this study were able to perform aerobic exercise, several participants might have attained the recommended activity level. Therefore, it is likely that the participants in the present study were relatively active stroke patients.

Because associations with physical activity vary according to different measurements of physical activity and the neighborhood environment as well as the physical activity domain observed, there was no consensus on the relationship between the two among older adults in a systematic review^10^. With regard to patients with stroke, we previously showed that the number of steps taken was significantly associated with perceived built environment attributes such as the presence of a sidewalk and access to recreational facilities in community-dwelling ambulatory patients with stroke^9^. However, only one study has investigated the relationship between physical activity and neighborhood walkability attributes using the Walk Score in patients with stroke. Recently, Miller et al. examined the role of social and physical environmental factors including walkability in explaining walking activity, measured in average steps per day, in patients with walkable stroke^16^. They showed that working and living in a less socioeconomically deprived neighborhood, rather than walkability, were related to the daily step activity. Their mean Walk Scores were lower than ours (33.0 ± 28.0 vs. 71.4 ± 17.2), but they were in line with the finding that physical activity was not associated with neighborhood walkability attributes. Thus, it can be stated that physical activity might not be associated with neighborhood walkability in patients with stroke who are able to walk.

In a community-dwelling older adult population, Chen et al. found a positive link between neighborhood walkability factors such as sidewalk availability and accelerometer-determined daily step count (not significant for total physical activity, light-intensity physical activity, MVPA, or long bouts of MVPA) in Taiwanese (mean age: 69.9 years, MVPA: 24.4 min/day)^15^. Amagasa et al. reported that Japanese people living in low-walkability areas accumulated more light-intensity physical activity and short-bout MVPA (mean age: 74 years, MVPA: 44.8 min/day)^19^. As indicated above, research in older Asian populations has not shown a consistent relationship between objectively measured physical activity such as the number of steps taken or intensity-based physical activity and neighborhood walkability attributes. Further studies are needed to clarify these two relationships in older adults or adults with disability including stroke.

In the present study, working status was significantly associated with the number of steps, and sex was significantly associated with MVPA in the patients with stroke. No other variables such as age, NIHSS, long-term care insurance, and walking speed were found to be associated with physical activity in either regression model, possibly because the study participants were limited to patients who were able to walk outside. Miller et al. reported that working status was statistically significantly associated with daily step count in a chronic stroke population, which was consistent with our results^16^. Unfortunately, we could not investigate how often the participants used cars or transportation and what type of work they did. The participants engaged in work might have walked to work or walked as a part of their work duties. It was not surprising that the female participants in the present study spent fewer minutes performing MVPA. Sex differences may affect intensity-based physical activity because of different roles in the home or society. However, Twardzik et al. found that the Walk Score had a positive association with accumulated daily time spent in MVPA, and this effect was not moderated by sex, age, or race within a national sample^20^. Additional studies should be conducted to examine the associations between objectively measured physical activity and neighborhood walkability attributes in patients with stroke by using sex-specific models.

This study has several strengths. It is the first study, to our knowledge, to examine associations between physical activity and neighborhood walkability attributes in community-dwelling ambulatory patients with stroke in an Asian area. We also objectively assessed physical activity according to the number of steps taken and MVPA using accelerometers. Furthermore, we evaluated neighborhood walkability attributes using the Walk Score to help eliminate the effects of a participant’s recall bias or perceived neighborhood environment.

The present study also has several limitations. First, we included only patients with stroke who were able to ambulate outside free of assistance, which limited the size of our study sample. Furthermore, the participants were limited to those living in walkable areas because of the small sample size. Thus, generalizability of the results of the present study requires caution. Second, we could not evaluate objectively measured sedentary behavior, which may also be affected by the impact of neighborhood walkability attributes^13,19^. Third, we did not evaluate other confounding factors related to physical activity such as details of physical function and psychological factors. This is evident from Table 2, which shows an adjusted R^2^ = 0.23 for the number of steps and 0.16 for MVPA, which means that only about 20% of the objectively measured physical activity was predicted by the factors we studied. Fourth, the present study was of a cross-sectional design. Kikuchi et al. examined the longitudinal association between neighborhood walkability attributes and 5-year changes in older adults’ physical activity^11^. Future longitudinal studies, such as that of the Kikuchi et al. study, may show a relationship between walkability and changes in physical activity in patients with stroke. Finally, as the Walk Score was originally scored based on walkability for the general population, it is possible that the present study might have biased the estimate of the effect of environment on walking for patients with stroke. Although the Walk Score was used in another study of patients with stroke^16^, it might be best to use it in conjunction with other assessments to evaluate built environments. To eliminate these problems, we plan to conduct a future study to address these limitations.

In conclusion, the results of the present study did not support the hypothesis that neighborhood walkability attributes would affect objectively measured physical activity in community-dwelling patients with stroke. If future studies reveal such a relationship, healthcare providers, policymakers, and community organizations should consider neighborhood walkability attributes in their efforts to promote physical activity in patients with stroke.

## Methods

### Participants

This cross-sectional study was conducted at a single hospital in Japan. Between August 2016 and November 2018, we screened consecutive outpatients with stroke who consented to the measurement of their physical activity at Itami Kousei Neurosurgical Hospital.

The inclusion criteria were a previous history of stroke and the ability to ambulate outside free of assistance. Exclusion criteria were patients with dementia or aphasia as evaluated by their primary care physician and those with a modified Rankin Scale^21^ score > 3 (moderate to severe disability conditions that require assistance with walking and physical demands) due to diseases other than stroke.

This study was approved by the research ethics committee of Kobe University Graduate School of Health Sciences (approval no. 690). Informed consent was obtained from all participants.

### Clinical characteristics

We evaluated participants’ clinical characteristics, including age, sex, body mass index (BMI), National Institutes of Stroke Scale (NIHSS)^22,23^, time since stroke, living situation, working status, whether they received long-term care insurance, and walking speed. The NIHSS is a valid, reliable, and reproducible scale for assessing neurologic severity, and it is also widely used in the clinical setting for stroke^23^. It is scored from 0 to 42 points, with lower scores indicating fewer neurological symptoms and higher scores indicating more severe symptoms. Walking speed was calculated as the result of a 10-m walking test^24^. Speed was derived from timing the patient’s walking over 10 m with a stopwatch by a physical therapist. Measurements were taken over the middle 10 m of a 14-m walkway. Patients were instructed to walk at a comfortable speed. Afterwards, walking speed was calculated as 10 m/time required in seconds by a physical therapist.

### Physical activity measurement

Participants were asked to wear an accelerometer, a Fitbit One (Fitbit, Inc., San Francisco, CA, USA), on their waist belt 24 h/day for at least 10 days except when changing clothes or bathing. The objectively measured physical activity included the number of steps taken and the duration of MVPA, which was collected in 60-s epochs^25^. We used 7 days of continuous data to determine objectively measured physical activity in the present study. We calculated the average number of steps (steps/day) and time spent in MVPA (min/day). MVPA was calculated by the sum of MVPA time spent at greater than 3 metabolic equivalent^26^. Participants could monitor physical activity in real time by manipulating the screen of the Fitbit device. During physical activity measurement, participants did not receive any kind of feedback from the physical therapist.

### Neighborhood walkability attributes

We evaluated neighborhood walkability attributes with the Walk Score^27^. The Walk Score is determined through a free, publicly accessible website that uses a geographically based algorithm to provide an estimate of neighborhood walkability attributes. The result is normalized to produce a score from 0 to 100, with a lower value indicating less walkability and a higher value indicating the most walkability. The values from 0 to 49 represent living in a “car-dependent” area, values from 50 to 69 represent living in a “somewhat walkable” area, values from 70 to 89 represent living in a “very walkable” area, and values from 90 to 100 represent a “walker’s paradise” area. The Walk Score has been used to evaluate the composite measure of neighborhood walkability in some Japanese studies^12–14^ and is a valid measure of neighborhood walkability attributes in Japan^12^. Koohsari et al. indicated that there were significant positive associations between the Walk Score and objectively calculated walkable built environment attributes in Japanese neighborhoods^12^.

### Statistical analysis

The results are shown as mean ± SD or as ordinal variables and counts (%) for categorical variables. Multiple linear regression analyses were used to determine the associations between objectively measured physical activity and neighborhood walkability attributes. Number of steps and MVPA were the respective dependent variables, and Walk Score was the independent variable. We changed the Walk Score to a categorical variable based on the quartiles of the Walk Score value (Q1: least walkable; Q2, Q3, Q4: most walkable). To adjust for confounding factors in the multiple linear regression analyses, clinical characteristics with a p value of < 0.05 in the univariate analysis for number of steps or MVPA were included as covariates. A p value of < 0.05 was considered to indicate statistical significance. Statistical analyses were performed with IBM SPSS ver. 25.0 statistical software (IBM SPSS Japan, Inc., Tokyo, Japan).

### Ethics declarations

The present study procedures were conducted in accordance with the Declaration of Helsinki. The present study was approved by the research ethics committee of Kobe University Graduate School of Health Sciences (approval no. 690). Informed consent was obtained from all participants.

## Data Availability

All relevant data are present within the paper. If additional data or permission to use the data is needed, for example, for use in a meta-analysis, it can be made available from the corresponding author for researchers who meet the criteria for access to confidential data.
